# Imbalanced Lignin Biosynthesis Promotes the Sexual Reproduction of Homothallic Oomycete Pathogens

**DOI:** 10.1371/journal.ppat.1000264

**Published:** 2009-01-16

**Authors:** Michaël Quentin, Valérie Allasia, Anthony Pegard, Florent Allais, Paul-Henri Ducrot, Bruno Favery, Caroline Levis, Sophie Martinet, Clarissa Masur, Michel Ponchet, Dominique Roby, Nikolaus L. Schlaich, Lise Jouanin, Harald Keller

**Affiliations:** 1 Unité Mixte de Recherches Interactions Biotiques et Santé Végétale, INRA-CNRS-UNS, Sophia Antipolis, France; 2 Unité de Chimie Biologique, INRA, Versailles, France; 3 Unité de Phytopathologie et Méthodologies de la Détection, INRA, Versailles, France; 4 Institut Bio III Pflanzenphysiologie, RWTH Aachen University, Aachen, Germany; 5 Laboratoire des Interactions Plantes-Microorganismes (LIPM), UMR INRA-CNRS, Castanet-Tolosan, France; 6 Laboratoire de Biologie Cellulaire, INRA, IJPB, Versailles, France; The University of North Carolina at Chapel Hill, United States of America

## Abstract

Lignin is incorporated into plant cell walls to maintain plant architecture and to ensure long-distance water transport. Lignin composition affects the industrial value of plant material for forage, wood and paper production, and biofuel technologies. Industrial demands have resulted in an increase in the use of genetic engineering to modify lignified plant cell wall composition. However, the interaction of the resulting plants with the environment must be analyzed carefully to ensure that there are no undesirable side effects of lignin modification. We show here that *Arabidopsis thaliana* mutants with impaired 5-hydroxyguaiacyl *O*-methyltransferase (known as caffeate *O*-methyltransferase; COMT) function were more susceptible to various bacterial and fungal pathogens. Unexpectedly, asexual sporulation of the downy mildew pathogen, *Hyaloperonospora arabidopsidis*, was impaired on these mutants. Enhanced resistance to downy mildew was not correlated with increased plant defense responses in *comt1* mutants but coincided with a higher frequency of oomycete sexual reproduction within mutant tissues. *Comt1* mutants but not wild-type *Arabidopsis* accumulated soluble 2-*O*-5-hydroxyferuloyl-l-malate. The compound weakened mycelium vigor and promoted sexual oomycete reproduction when applied to a homothallic oomycete *in vitro*. These findings suggested that the accumulation of 2-*O*-5-hydroxyferuloyl-l-malate accounted for the observed *comt1* mutant phenotypes during the interaction with *H. arabidopsidis*. Taken together, our study shows that an artificial downregulation of *COMT* can drastically alter the interaction of a plant with the biotic environment.

## Author Summary

Lignin is an essential component of wood and the second most abundant terrestrial biopolymer. Plants synthesize lignin to strengthen cell walls and to resist pathogen attack. Because the technological value of plants is determined by the amount and composition of lignin, genetic engineering approaches frequently aim at altering these parameters. However, data showing how plants with modified lignin content interact with the biotic environment are still scarce. This study is the first of its kind to evaluate how a model plant, which was mutated for a key enzyme in lignin biosynthesis, interacts with pathogens from different kingdoms. We found that the mutants were generally more susceptible to bacteria and fungi. Additionally, the mutation altered the plant physiology in a manner that elevated sexual reproduction of an oomycete pathogen. Oospores resulting from this mode of reproduction are the most persisting propagules of this pathogen. Their elevated formation correlated with the accumulation of a soluble phenylpropanoid derivative in the mutants, which we synthesized and found to be able to stimulate sexual oomycete reproduction *in vitro*. Our study indicates that interfering with lignin composition may drastically alter the outcome of plant–pathogen interactions.

## Introduction

**Figure 1:**
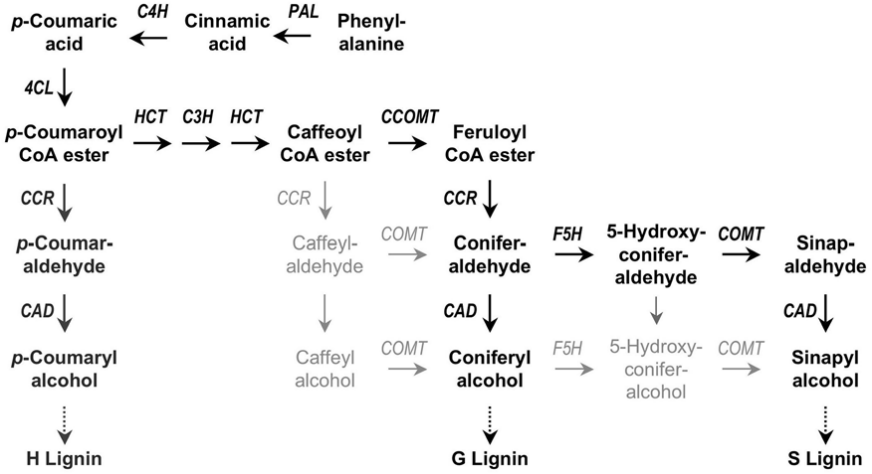
Simplified scheme for monolignol synthesis. The main pathway in dicotyledonous plants is highlighted in black, involving phenylalanine ammonia-lyase (*PAL*), cinnamate 4-hydroxylase (*C4H*), 4-coumarate CoA ligase (*4CL*), *p*-hydroxycinnamoyl-CoA: quinate shikimate *p*-hydroxycinnamoyltransferase (HCT), *p*-coumarate 3-hydroxylase (*C3H*), caffeoyl-CoA *O*-methyltransferase (*CCOMT*), hydroxycinnamyl-CoA reductase (*CCR*), ferulate 5-hydroxylase (*F5H*), caffeate *O*-methyltransferase (COMT), and cinnamyl alcohol dehydrogenase (*CAD*). Alternate pathways are in light grey. H subunits are only minor lignin components in dicots. Adapted from [1],[4].

Lignin accounts for about 30% of the organic carbon in the biosphere and is the second most abundant terrestrial biopolymer after cellulose [1]. Lignin fortifies plant cell walls, is the essential composite of wood, and allows terrestrial plants to gain size and volume. This polymer renders the walls of water-conducting cells impermeable, making it possible for the xylem vessels to transport water [2]. Plants with high lignin content are less accessible to microbes and insect herbivores, and the incorporation of this polymer into cell walls is an important mechanism of plant defense against pathogen attack [3]. Lignin is a complex aromatic heteropolymer composed of the three phenylalanine-derived monolignols, *p*-coumaryl-, coniferyl-, and sinapyl alcohol [1],[4] (Figure 1). Polymers composed of these monolignols are referred to as *p*-hydroxyphenyl (H), guaiacyl (G), and syringyl (S) lignin, respectively. Dicotyledonous plants contain mostly G and S lignin, whereas monocotyledonous plants contain H, G, and S lignin [5]. The amount of lignin and the proportions of the H, G, and S subunits within the lignocellulosic biomass determine the technological value of the raw plant material for forage, biofuel distillation, wood, or paper production. Attempts to modify lignin composition have been a major focus of research for decades, with the first mutant described as early as 1935 [6]. The *brown midrib-3* (*bm3*) maize mutant has a lower S-lignin content than normal maize [7], due to the downregulation of caffeate *O*-methyltransferase (COMT) activity [8] (Figure 1). COMT has since been targeted repeatedly in efforts to improve the technological quality of plant material by transgenic approaches. The downregulation of COMT activity leads to a decrease in the proportion of S-lignin, with no major change in overall lignin content [9]. As previously reported for *bm3* maize [10], an increase in the G/S lignin ratio generally seems to improve the digestibility of transgenic forage [11]. Poplar trees in which *COMT* expression has been silenced have been shown to generate higher pulp yields, although the lignins they contain are less amenable to industrial degradation [12]. In field trials, the transgenic trees grew normally and displayed no obvious physiological alterations [13]. However, studies investigating the side effects of changes in *COMT* gene expression in mutant and transgenic plants remain scarce, although it was shown 30 years ago that the *bm3* mutation decreases vascular integrity and stalk robustness in maize [14]. Even less is known about the outcome of biotic interactions involving plants with genetically modified *COMT* expression and S-lignin content. *Arabidopsis thaliana* appears to be an excellent tool for addressing this issue. This plant has a typical dicotyledonous lignification pattern [15], the *COMT* gene has been clearly identified [16], tools for functional analyses have been generated, and *A. thaliana* has been established as a host for a large number of microorganisms. In this study, we used wild-type *A. thaliana* and mutant *A. thaliana* lines with inactivated *COMT1* for analyzing their phenotypes of interaction with the bacterial pathogens *Xanthomonas campestris* pv. *campestris* and *Pseudomonas syringae* pv. *tomato*, the fungal pathogens *Alternaria brassicicola*, *Blumeria graminis*, and *Botrytis cinerea*, the oomycete *Hyaloperonospora arabidopsidis* (formerly *H. parasitica*), and the root-knot nematode *Meloidogyne incognita*. The *comt1a* mutant was found to have much lower levels of S-lignin than the wild type, although its overall lignin content was similar [15]. Knockout of *comt1* led to a strong decrease in soluble 2-*O*-sinapoyl-L-malate (sinapoyl malate) levels and the accumulation of 2-*O*-5-hydroxyferuloyl-L-malate (hydroxyferuloyl malate), which is not detectable in wild-type plants ([4],[15] and this study). We paid particular attention to interactions with the oomycete *H. arabidopsidis*, because *comt1* mutants displayed an unexpected phenotype when inoculated with this pathogen. Oomycetes have devastating effects on crops, forests, and natural ecosystems, and there are currently no efficient methods for their control [17]. These fungus-like pathogens have unique physiological characteristics and can undergo vegetative or sexual reproduction. Asexual reproduction leads to the creation of clonal populations well adapted to a given host and environment. Sexual reproduction can occur in a single strain, when the species is homothallic. In heterothallic species, two strains of opposite mating types are required for fertilization. In both homothallic and heterothallic species, fertilization results in thick-walled zygotes called oospores, which are produced in infected plant tissue, and released into the soil as the plant tissue degrades. Oospores are highly resistant against environmental influences and can persist in the soil for several years [18]. Sexual reproduction is frequently initiated in response to selective pressure from the environment [19]. The findings presented here indicate that mutations in *COMT1* alter the plant physiology in a way that stimulates the oomycete to reproduce sexually. Risk assessments for plants with modified lignin biosynthesis should, therefore, go for quantitative studies of the interaction with the biotic environment, and beyond.

## Results

### Characteristics of *comt1* mutant and transgenic plants

Two allelic mutant lines for *COMT1* in the Wassilewskija (WS) background were identified from the Versailles *Arabidopsis* insertion mutant collection [20]. The *comt1a* and *comt1b* mutants were homozygous for unique T-DNA insertions in the first exon and the first intron of the At5g54160 locus, respectively (Figure S1A). *Comt1a* has been shown to be a positive promoter-trap line expressing a functional *ß*-glucuronidase (*GUS*) gene under the control of the *COMT1* promoter [15]. *Comt1b* harbors the *GUS* gene as an in-frame insertion in the first intron (Figure S1A), providing GUS activity upon transcriptional activation of COMT1. For mutant phenotype complementation, a poplar *PtOMT1* cDNA was placed under the control of a constitutive promoter and transferred into the *comt1a* mutant. *PtOMT1* was used to avoid the silencing effects observed in *Arabidopsis* when the *COMT1* gene is overexpressed [15]. The complementation line, CpOMT14, had almost normal levels of COMT activity and S-lignin [15]. The exon-tagged *comt1a* mutant lacked the *COMT1* mRNA signal found in wild-type and CpOMT14 plants on reverse transcription-PCR analysis (RT-PCR, Figure S1B). The T-DNA insertion in *comt1b* did not lead to a full gene knock-out, but did decrease levels of *COMT1* mRNA (Figure S1B), which was correctly spliced and had a sequence identical to that of the wild-type *COMT1* mRNA (data not shown).

### Transcriptional activation of *COMT1*

**Figure 2:**
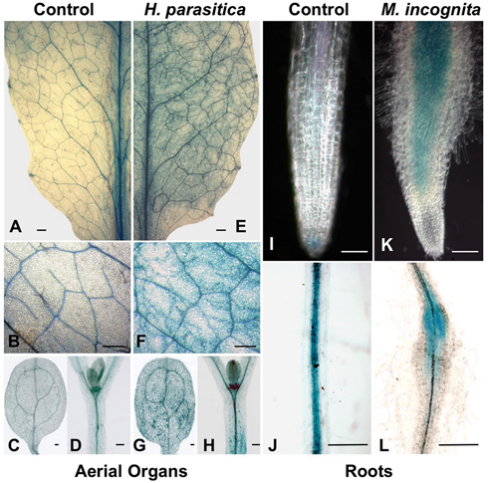
GUS histochemical analyses reporting pathogen-inducible transcriptional activation of *COMT1* in *A. thaliana*. (A–H) GUS activity in aerial organs, before (A–D), and 4 days post inoculation (dpi) with *H. arabidopsidis* (E–H). GUS staining occurred in the vascular system (A) and in veins (B) of adult leaves and cotyledons (C), as well as in leaf primordia (D), in non-infected plantlets. Upon infection, GUS staining extended to cells close to oomycete hyphae in the leaf lamina (E,F), in cotyledons (G), and in the hypocotyl (H). (I–L) *COMT1* expression in roots, before (I,J), and after inoculation with *M. incognita* (K,L). Constitutive GUS activity was found in columella root cap cells (I), and in the central cylinder (J). Upon inoculation, GUS staining extended to all cells of the swelling gall, 7 dpi (K). After 21 days, GUS activity was found in nematode-induced giant cells and neighboring parenchyma cells of the feeding site (L). Scale bars represent 1 mm in (A,B,E,F), and 100 µm in (C,D,G–L).

The expression profile of *COMT1* was analyzed by GUS staining plant tissues from the promoter-trap lines *comt1a* and *comt1b*. Identical profiles of GUS activity were detected in tissues from both lines (data not shown). Under normal growth conditions, GUS activity was restricted to tissues undergoing lignification, such as the vascular systems within aerial organs (Figure 2A–D) and the root central cylinder (Figure 2J). Additional constitutive expression was observed within the distal focus [21] of cotyledons (Figure 2C) and developing young leaves, within leaf primordia (Figure 2D) and root cap columella cells (Figure 2I), suggesting that *COMT1* expression responds to venation patterning and auxin signaling [22]. Upon infection with the biotrophic oomycete leaf pathogen, *H. arabidopsidis*, *COMT1* expression extended to mesophyll parenchyma in leaves (Figure 2E and 2F), cotyledons (Figure 2G), and the hypocotyl (Figure 2H). Expression was restricted to cells close to invading hyphae (Figure 2F). In roots, the biotrophic root-knot nematode, *M. incognita*, invades cortex cells, migrates to the root tip, enters the central cylinder, and moves upward before settling and inducing the formation of a feeding site [23]. This nematode activated *COMT1* expression early in cells of the swelling gall (Figure 2K), in giant cells and surrounding dividing cells. *COMT1* expression was maintained in the mature root gall until 21 days after inoculation (Figure 2L), in both the giant cells and their neighboring cells. Thus, parenchyma cells from different plant organs did not express *COMT1* under normal growth conditions. However, the same cells responded to pathogen infection by local transcriptional activation of this gene.

### Susceptibility of a *comt1* mutant to microbial and nematode pathogens

We compared the susceptibility of wild-type plants and of the *comt1a* mutant to diverse pathogenic microorganisms, including fungi, bacteria, and nematodes (for details, see Protocol S1). We found the mutant significantly more susceptible to a strain of the necrotrophic fungus, *Botrytis cinerea* [24] (Figure S2A). Increased susceptibility was also observed for two other analyzed fungal pathogens, the necrotrophic *Alternaria brassicicola* [25] and the biotrophic *Blumeria graminis* f. sp. *hordei* (*Bgh*) [26] (Figure S2B and S2C). In addition, the *comt1* mutant was found to be significantly more susceptible to the xylem-colonizing systemic bacterial pathogen *Xanthomonas campestris* pathovar *campestris* (*Xcc*) [27], and the bacterial speck agent, *Pseudomonas syringae* pv. *tomato* (*Pst*) (Figure S3A and S3B). The conclusion from these experiments was that a mutation of *COMT1* generally weakens plant resistance to microbial pathogens. An exception from this observation was only found for the root-knot nematode *M. incognita*. Although the *M. incognita* infection activated *COMT1* (Figure 2), a gene knockout did not influence the mean number of galls established three weeks after inoculation. It did neither affect nematode ability to complete its life cycle, which was characterized by similar amounts of egg masses produced by *M. incognita* two months after inoculation in mutant and wild-type plants (Figure S2D).

### Susceptibility of *comt1* mutants to *H. arabidopsidis*

**Figure 3:**
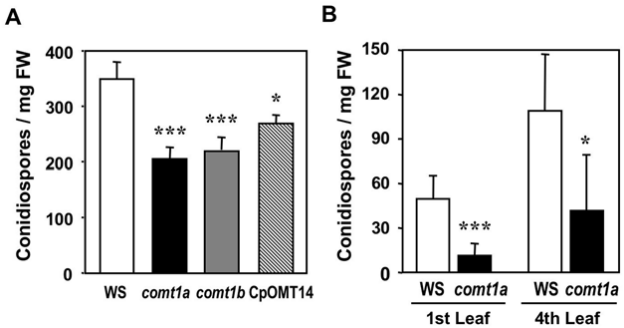
The mutation of *COMT1* affects asexual sporulation of *H. arabidopsidis*. (A) Sporulation of *H. arabidopsidis* isolate Emwa1 on cotyledons of *Arabidopsis* wild-type plantlets (WS), mutants (*comt1a* and *comt1b*), and complemented mutants (CpOMT14), 6 dpi. Conidiospores were harvested from 8 pooled plants and counted. The bars represent mean values±SD for 20 repetitions. (B) Sporulation of Emwa1 on the 1st and 4th fully developed leaf of 4 week-old *Arabidopsis*, 6 dpi. The bars represent mean values±SD from 10 individual leaves. All experiments were repeated three times and gave similar results. Statistically significant differences for values compared with the wild type were determined by Student's t-test (* P<0.01, ** P<0.001, *** P<0.0001).

Transcriptional activation of *COMT1* occurred as a consequence of oomycete infection (Figure 2). We therefore analyzed whether an inactivation of *COMT1* influences the interaction between *H. arabidopsidis* and *A. thaliana* under laboratory conditions. The virulent isolate Emwa1 completed its infection cycle on wild-type and *comt1* mutant plants, resulting in asexual reproduction and the formation of conidia. However, sporulation levels were 40 to 50% lower on *comt1* mutants than on wild-type plants (Figure 3A). This enhanced resistance phenotype of seedlings was partly complemented by expressing *PtOMT1* in the *comt1a* mutant background (Figure 3A), and was confirmed on adult plants, with true leaves from different rosette stages displaying significantly lower levels of *H. arabidopsidis* sporulation in the absence of COMT1 (Figure 3B).

We investigated whether the observed phenotype of *comt1* mutants resulted from a gain-of-function in defense signaling, by assessing constitutive and inducible responses before and after inoculation with *H. arabidopsidis*. The markers used for the activation of salicylic acid (SA)- and jasmonic acid (JA)-dependent responses were the transcriptional activation of *PR-1a* and *PDF1.2b*, respectively. In quantitative RT-PCR experiments, *PR-1a* mRNA was undetectable in untreated wild-type and mutant plants. *PDF1.2b* transcripts were present at similar, low levels in both lines tested in the absence of inoculation. The accumulation of transcripts from both genes was induced by the pathogen, to similar levels in wild-type and mutant plants (Figure S4). The enhanced resistance phenotype of *comt* mutants thus appeared not to be dependent on SA and JA-dependent defense signaling pathways in *Arabidopsis*.

### Development of the oomycete pathogen within *comt* mutants

**Figure 4:**
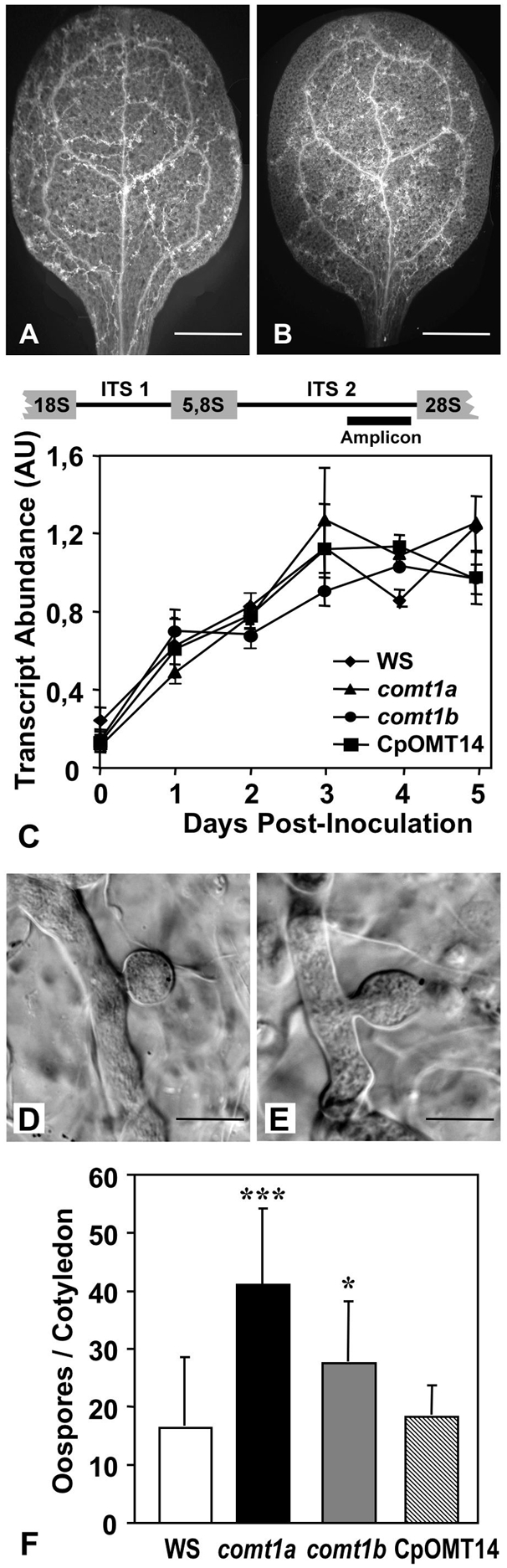
The mutation of *COMT1* impairs haustorium stability, and favors sexual reproduction. (A,B) General view of the calcofluor-stained *H. arabidopsidis* hyphal network in cotyledons of wild-type *Arabidopsis* (A) and the *comt1a* mutant (B), 3 dpi. (C) Quantification of the *H. arabidopsidis ITS2* amplicon as a marker for oomycete biomass. RT-PCR was performed using total RNA, which was extracted at different time points post inoculation from infected cotyledons of *Arabidopsis* wild-type plants (WS), mutants (*comt1a* and *comt1b*), and complemented mutants (CpOMT14). RNA abundance is expressed as the signal intensity ratio between *ITS2* and *OXA1* amplicons. Displayed are means±SD from 2 repetitions of the experiment. (D,E) Shape of haustoria in host cells from a wild-type (D) and a *comt1a* mutant (E) plant. Shown are differential interference contrast micrographs of calcofluor-stained hyphae and haustoria within infected tissues, 3 dpi. Scale bars represent 5 µm. (F) Frequency of sexual reproduction of *H. arabidopsidis* within infected tissues of *Arabidopsis* wild-type plants, mutants, and the complemented mutant, expressed as the number of oospores formed per infected cotyledon. The bars represent mean values±SD from 20 individual cotyledons. The experiment was repeated three times and gave similar results. Statistically significant differences for values compared with the wild type were determined by Student's t-test (* P<0.01, *** P<0.0001).

Following the inoculation of wild-type *A. thaliana*, *H. arabidopsidis* spores germinate on leaf surfaces and form appressoria, enabling the penetration pegs to overcome the cuticle. Once inside the leaf, the hyphae grow intercellular, branch, and establish a filamentous network spanning the entire infected leaf three to four days after the onset of infection (Figure 4A). The growing hyphae locally digest the cell walls of almost all the plant cells they come into contact with, leading to invagination of the host plasma membrane and the formation of intracellular bulbous structures called haustoria (Figure 4D). Haustoria are feeding sites required for the biotrophic lifestyle of the oomycete. Four to six days after inoculation under laboratory conditions, the hyphae use stomatal openings to form conidiophores on the leaf surface and initiate asexual reproduction [28]. In *comt1* mutant lines, the initial infection process was identical to that in wild-type plants. *H. arabidopsidis* penetrated, formed a filamentous network of branched hyphae (Figure 4B), and developed haustoria within host cells. We quantified the development of the oomycete in wild-type and mutant *Arabidopsis*, by performing RT-PCR with gene-specific primers to generate an amplicon within the intergenic transcribed spacer (ITS2) of *H. arabidopsidis* ribosomal RNA (Figure 4C). These experiments showed that the oomycete expanded similarly in wild-type and mutant plants (Figure 4C), indicating that hyphal growth and branching were not affected in the mutants. However, microscopic analyses of haustoria indicated that the structure of the feeding sites was less stable in *comt1* mutant plant cells than in wild-type cells. Disintegration of the haustorium was observed in mutant cells (Figure 4E) but never in the wild-type background. Even more strikingly, the frequency of sexual reproduction was found to be higher in *comt1* mutants, with a significantly larger number of oospores within mutant tissues than within wild-type tissues (Figure 4F). This phenotype was almost complemented in the CpOMT14 line (Figure 4F). Thus, the lower level of conidiospore formation in *comt1* mutants (Figure 3) coincided with a higher frequency of sexual reproduction.

### Metabolic disequilibrium in *comt1* mutants, and its impact on oomycetes

Plant cells store excess monolignol precursors not incorporated into lignin as soluble esters. In the leaves and cotyledons of *A. thaliana*, the major accumulating soluble hydroxycinnamate ester is sinapoyl malate (SM) [29]. In the *comt1a* mutant, SM levels are much lower, with these mutants instead accumulating hydroxyferuloyl malate (OH-FM), which is a derivative of the COMT1 substrate, 5-hydroxyconiferaldehyde [4],[15].

**Figure 5:**
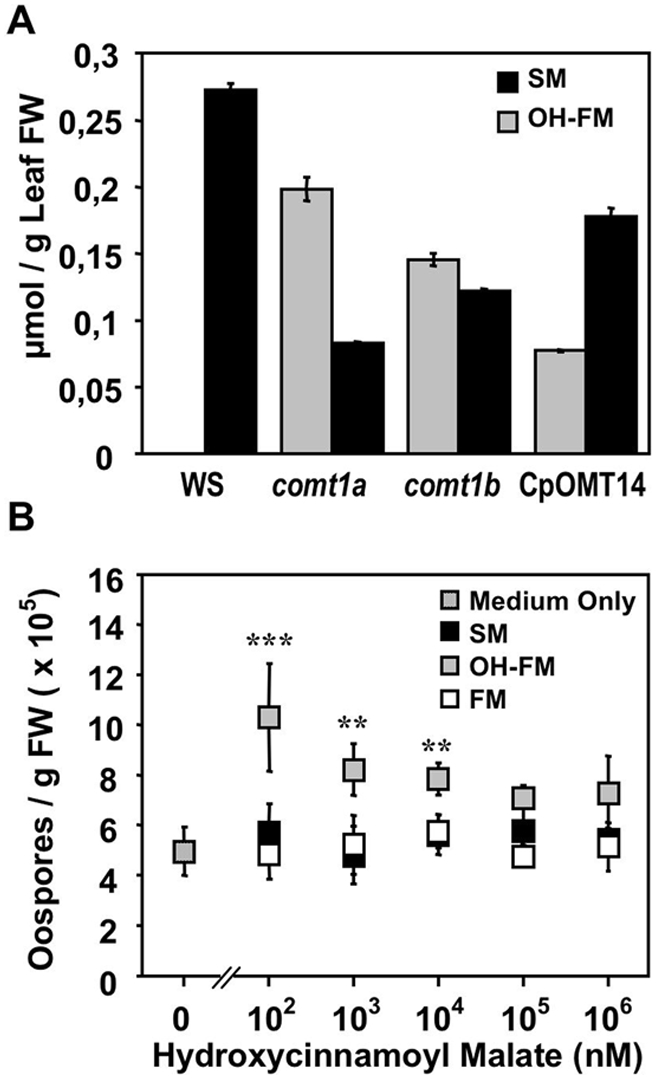
2-*O*-5-hydoxyferuloyl-L-malate accumulates in *comt1* mutants, and promotes oomycete sexual reproduction *in vitro*. (A) Quantification of sinapoyl malate (SM) and 5-hydroxyferuloyl malate (OH-FM) in methanolic extracts of 4 week-old plantlets from the different *A. thaliana* lines. The compounds were quantified by HPLC referring to synthetic standards. The bars represent mean values±SD from 3 experiments. (B) Effect of different concentrations of SM, OH-FM, and feruloyl malate (FM) on sexual reproduction of *Phytophthora cactorum* isolate 723 *in vitro*. Displayed are means±SD from 4 repetitions. Statistically significant differences for OH-FM values compared with values in the absence of the compound were determined by Student's t-test (** P<0.001, *** P<0.0001). FW = fresh weight.

We quantified the accumulation of hydroxycinnamoyl malate esters in the various lines used in this study and evaluated the biological activities of these compounds, by synthesizing SM, OH-FM, and 2-*O*-feruloyl-L-malate (feruloyl malate; FM) (see Protocol S2, and Figure S5). On reverse-phase high-performance liquid chromatography (HPLC) of methanolic extracts from four-week-old plantlets, FM was not detected in any of the *Arabidopsis* lines. Wild-type plants accumulated soluble SM to a concentration of about 280 nmol/g fresh weight and OH-FM was not detectable in these plants (Figure 5A). By contrast, the mutant lines *comt1a* and *comt1b* accumulated OH-FM to concentrations of 200 nmol and 150 nmol/g fresh weight, respectively, whereas SM levels were about 70% lower than wild-type levels in *comt1a*, and 55% lower in *comt1b* (Figure 5A and Figure S3). SM was again the main compound detected in seedlings from the complemented mutant line CpOMT14 (Figure 5A). The *COMT1* mutation modified the SM/OH-FM ratio, but did not modify total hydroxycinnamoyl malate ester concentrations, which appeared to be similar in plants from all lines.

The correlation between the accumulation of soluble OH-FM (Figure 5A) and the frequency of sexual reproduction (Figure 4F) in the *comt1* mutant lines led us to analyze whether this compound was able to stimulate oospore formation. We thus developed an *in vitro* assay for the sexual reproduction of another homothallic oomycete plant pathogen, *Phytophthora cactorum*. Using *P. cactorum* zoopores, we synchronized the age and density of hyphae in titer plate wells before adding OH-FM, SM, or FM at various concentrations. In the absence of these compounds, *P. cactorum* produced about 5,000 oospores (per g fresh weight of mycelium). The addition of SM or FM did not alter the frequency of sexual reproduction of the oomycete (Figure 5B). In contrast, supplementation of the medium with OH-FM significantly stimulated sexual reproduction, and the number of oospores doubled when OH-FM was added to concentrations of about 0.1 µM (Figure 5B). Beyond 0.1 mM, the effect of OH-FM on sexual reproduction was less obvious. At these concentrations, OH-FM appeared to influence the integrity of the hyphal network, and the mycelium developing in the presence of the compound became frail. These findings strongly suggest that the accumulation of OH-FM can account for the greater sexual activity of *H. arabidopsidis* in *comt1* mutants. However, OH-FM did not stimulate the sexual reproduction of individual mating types of the heterothallic oomycete, *P. parasitica* (data not shown). OH-FM therefore cannot replace an oomycete mating hormone.

## Discussion

In recent decades, lignin has become a privileged target for genetic engineering approaches aiming to increase the industrial processing efficiency of plant biomass. However, the pros and cons of the use of such transgenic plants in open-field situations have only recently begun to be explored [30]–[32]. Systems biology approaches have been used to investigate the extent to which a single engineered gene encoding a protein involved in the lignin biosynthesis pathway interferes with other metabolic pathways, and the extent to which it modifies interactions between the modified plant and its environment [33]. The downregulation of *CAD* and *CCR* (Figure 1) in tobacco has been shown to affect not only lignin biosynthesis, but also primary metabolism, stress metabolism, and photorespiration [34]. In poplar, the downregulation of *CCR* affects the overall metabolism and structure of cell wall polymers [35]. This enzyme also directly regulates pathogen defense signaling in rice [3], indicating that lignin biosynthesis plays a more subtle role in plant defense responses against herbivores [36] and microbes [37] than simply constituting a physical barrier.

This report provides the first large-scale analysis of the role of *COMT* in interactions between the plant and its biotic environment. We found that the transcriptional activation of *COMT1* in parenchyma tissues from roots and aerial organs of *Arabidopsis* was stimulated by pathogen infection. *COMT1* expression was shown before to be enhanced upon *Arabidopsis* leaf infiltration with either the nonhost bacterium *P. syringae* pv. *phaseolicola* [38], bacterial flagellin (flg22) [38],[39], or harpin (HrpZ) [38],[40], or with the necrosis-inducing *Phytophthora* protein 1 (NPP1) [38],[41]. The transcription of *COMT1* appears thus to correlate with the onset of the plant's pathogen-associated molecular pattern (PAMP)-triggered immune (PTI) response [42]. In the *Arabidopsis* leaf mesophyll, we found an accumulation of toluidine blue-stainable, lignin-like deposits in host cells that surround *H. arabidopsidis* hyphae (data not shown). Taken together, we suppose that the observed *COMT1* promoter activation in response to infection reflects an onset of PTI within host cells and an attempt to reinforce wall rigidity through lignification. Consequently, we found that *COMT1* downregulation increased susceptibility to at least three fungal and two bacterial pathogens. Mutants were significantly more susceptible to a moderately aggressive strain of the necrotrophic fungus, *B. cinerea*, but not to a highly virulent isolate. These findings indicate that COMT1 contributes to limiting the spread of manageable strains of the fungus. The *comt1a* mutant was also more susceptible to another necrotrophic fungal pathogen, *Alternaria brassicicola*. This fungus causes black spot disease on almost all cultivated *Brassica* species including broccoli, cabbage, canola and mustard. It is of worldwide economic importance, because it reduces crop yields and the quality of canola oil [43]. From a human health perspective, *Alternaria brassicicola* belongs to a genus of fungi considered one of the most potent sources of mold-derived allergens [44]. The resistance of *A. thaliana* to this fungus requires the phytoalexin camalexin and the signaling molecule JA, but is independent of SA [25]. The downregulation of *COMT1* weakened the JA-dependent defenses of *Arabidopsis* against this pathogen. Unlike *B. cinerea* and *Alternaria brassicicola*, *Bgh* is a biotrophic fungal pathogen causing powdery mildew on barley. *Arabidopsis* is a nonhost for this pathogen, and SA-dependent defense responses are activated rapidly when the pathogen attempts to penetrate [26]. The higher proportion of successful infections in the mutant indicated that the mutation of *COMT1* weakens this penetration resistance of *A. thaliana*.

Mutants were also more susceptible to the bacterial pathogens *Pst* carrying *avrPphB*, and *Xcc*. AvrPphB is recognized by the corresponding resistance gene product, RPS5, which is present in the WS background, but absent in the *Ler* background [45]. Plants of these two ecotypes are thus resistant and susceptible, respectively, to *Pst avrPphB*. The observation that *COMT1* downregulation renders WS as susceptible as *Ler* indicates that the gene plays also an important role in *avrPphB*-mediated resistance to the bacterial pathogen.

Two exceptions to the trend of *comt1a* mutants being generally more susceptible to pathogens were observed after inoculation with the root-knot nematode, *M. incognita*, and the biotrophic oomycete pathogen, *H. arabidopsidis*. *M. incognita* triggers the transcriptional activation of *COMT1*, but knocking out this gene had no effect on disease establishment (gall formation), or nematode development (egg masses). However, all the *Arabidopsis* ecotypes analyzed to date are susceptible to *M. incognita*. Variety-specific differences in defense and resistance influencing nematode development, such as those within the Solanaceae [46], are not known for *A. thaliana*. We therefore cannot exclude the possibility that *COMT1* may affect resistance phenotypes in other plant species. In this context, it has been reported that a downregulation of the *COMT1* ortholog in tobacco [47], leads to increased *M. incognita* reproduction [48].

The obligate oomycete pathogen *H. parasitica* causes downy mildew disease on agronomically important *Brassicaceae*, such as rapeseed and cabbage. Its relative, *H. arabidopsidis* only infects *A. thaliana* in clear gene-for-gene relationships and is nonpathogenic on other crucifers tested [49]–[51]. WS harbors genes from the *RPP1* group (*RPP1-WsA*, *RPP1-WsB*, and *RPP1-WsC*), which confer resistance to the *H. arabidopsidis* isolate Noco2, but not to Emwa1 [50]. These two isolates thus give rise to genetically incompatible and compatible interactions, respectively, with WS. In *Arabidopsis* seedlings inoculated with Noco2, *comt1* mutants displayed the same resistance phenotype as WS, with no hyphal development detectable in any of the lines tested (data not shown). COMT1 is thus not a key enzyme for the genetic resistance of *Arabidopsis* to oomycetes. However, the virulent *H. arabidopsidis* isolate Emwa1 produced significantly fewer conidiospores on *COMT1* mutants than on wild-type plants. According to the generally accepted criterion for analyzing plant susceptibility to this oomycete [52], *comt1* mutants were thus more resistant. To date, several *Arabidopsis* genes have been identified, which are required for full susceptibility to biotrophic fungal and oomycete pathogens, and which confer, when inactivated, increased resistance phenotypes to mutants. Vogel and coworkers identified 26 recessive *powdery mildew resistance* (*pmr*) mutants in a genetic screen [53]. *PMR6* codes for a pectate lyase [54], and *PMR2* is the *Arabidopsis* ortholog of the barley *mlo* gene, *Atmlo2* [55]. The MLO protein is required for successful entry of *Bgh* into the host cell [56]. Both *pmr6* and *Atmlo2* mutants were not affected in susceptibility to *H. arabidopsidis* [54],[55], indicating that the downy mildew does not require the host cell pectate lyase and MLO for successful infection. However, another *pmr* mutant, *pmr4* (or *gsl5*) is more resistant to both powdery- and downy mildews [53]. *PMR4* codes for a callose synthase, which seems to be involved in yet unknown functions required for functional haustoria formation [57], and which negatively regulates SA defense signaling in the plant [58]. Since, six additional *Arabidopsis* loci were identified that confer, when mutated, resistance to *H. arabidopsidis* [59]. For three of the *downy mildew resistant* (*dmr*) mutants, enhanced resistance was correlated with a constitutive expression of the SA-dependent *PR-1a* gene [59]. Of the remaining *DMR* genes, *DMR6* was identified to code for a 2-oxoglutarate (2OG)-Fe(II) oxygenase of unknown function [60]. The role of DMR6 for disease susceptibility is not yet known, but it also appears to be a negative regulator of defense gene activation [60]. Here, we showed that the increased resistance of *comt1* mutants was not correlated with a change in *PR-1a* and *PDF1.2* gene expression, when compared to wild-type plants. The inactivation of *COMT1* appears, therefore, not to interfere with SA- and JA-dependent defense signaling pathways.

The absence of COMT1 did not impair intercellular hyphal growth and branching, and did not interfere with overall oomycete biomass development in mutant plant tissues. However, *H. arabidopsidis* interacting with mutant plants displayed haustorial instability and enhanced sexual reproduction as two additional phenotypes to reduced asexual sporulation. We found that the occurrence of these phenotypes correlated with a metabolic difference between *comt1* mutants and wild-type plants, i.e. lower levels of SM within mutants and accumulation of OH-FM instead. Synthetic OH-FM promoted the sexual reproduction of *P. cactorum in vitro*, whereas the closely related derivative SM, which was present in large amounts in wild-type *Arabidopsis*, and synthetic FM did not. Moreover, high OH-FM concentrations decreased the mechanical resistance of the *P. cactorum* hyphal network *in vitro*, indicating that the compound is toxic for oomycetes and likely responsible for the observed haustorial instability phenotype of *H. arabidopsidis* in *comt1* mutants. These findings make OH-FM accumulation a potential candidate cause for the phenotypes we observed during the interaction between *comt1* mutants and *H. arabidopsidis*. In this context, it might be possible that the observed transcriptional activation of *COMT1* in host cells close to downy mildew hyphae has another reason than the above discussed stimulated lignification defense. In a hypothetical scenario, an *H. arabidopsidis* virulence function might promote host *COMT1* expression, in order to prevent plant cells harboring oomycete haustoria from any accumulation of detrimental OH-FM. However, OH-FM was not detectable in wild-type *Arabidopsis* by the means we used for the present study. To prove the hypothetical scenario, more sensitive detection methods for OH-FM need to be developed to compare the accumulation of microquantities of the compound in living cells harboring or not haustoria.

In conclusion, this study demonstrates that manipulating the expression of single genes within the monolignol biosynthetic pathway may affect the interaction of engineered plants with the biotic environment. COMT has been a key target for such manipulation in the past [13]. The downregulation of *COMT* in agronomically important plants may affect susceptibility to fungal and bacterial attack in a similar manner, as shown here for *A. thaliana*. The metabolic disequilibrium generated by *COMT1* knockout in *Arabidopsis* probably also creates a selective pressure in other crops that forces oomycete pathogens to undergo sexual reproduction. Sexual reproduction is a source of genetic variation even in homothallic oomycetes [61],[62]. Furthermore, oospores persist in the soil [63],[64] and are insensitive to fungicides [65], and thus make disease control difficult. The stimulation of oomycete sexual reproduction in genetically engineered COMT plants may, therefore, lead to the evolution of novel infection traits.

## Materials and Methods

### Plant material and growth conditions

All *Arabidopsis* lines used for the experiments were from the Wassilewskija (WS) genetic background. The *comt1a* mutant, previously named *Atomt1* [15] and *comt1* [4], and the complemented line CpOMT14 have been described before [15]. The *comt1b* mutant from the Versailles T-DNA insertion collection [18] was obtained from Dr. Laurent Nussaume (CEA, Cadarache, France). Analysis of the T-DNA flanking region within *comt1b* was performed by sequencing the amplicon obtained with primer pair 3 and 4 on genomic DNA as the template (Figure S1). Plants were grown in sand supplemented with MS medium in growth chambers at 20°C with a 12 h photoperiod.

### Pathogen assays

*H. arabidopsidis* isolates Emwa1 and Noco2 were obtained from Dr. Jane Parker (MPIZ, Cologne, Germany), and transferred weekly onto the genetically susceptible *Arabidopsis* accessions WS and Columbia (Col), respectively, as described [66]. For infection, 10-day-old plants were spray-inoculated to saturation with a spore suspension of 40,000 spores/ml. Plants were kept in a growth cabinet at 16°C for 3 d with a 16 h photoperiod. Sporulation was then induced by spraying plants with water, and keeping them for 48 hours under high humidity. To evaluate conidiospore production, pools of 8 plants were harvested in 1 ml of water. After vortexing, the amount of liberated spores was determined with a hemocytometer. To evaluate oospore production, inoculated cotyledons were destained in 80% ethanol, mounted on glass slides, and analyzed by microscopy.

For nematode infection, *A. thaliana* were grown *in vitro* on MS medium containing 1% sucrose and 0.7% plant cell culture-tested agar (Sigma-Aldrich). One hundred surface-sterilized freshly hatched *M. incognita* J2 larvae were added to each 2-week-old seedling, as described [67]. The plates were kept at 20°C with a 16 h photoperiod.

Pathogenicity assays with *B. cinerea*, *A. brassicicola*, *B. graminis*, *P. syringae*, and *X. campestris* were performed as described in Protocol S1.

### Histochemical analyses

GUS activity in *comt1a* mutants was analyzed histochemically [68]. For the observation of oomycete development in infected tissues, cotyledons were fixed in 0.1 M glutaraldehyde and 1.5 M formaldehyde in phosphate buffer, bleached in a series of increasing ethanol concentrations, and stained with 0.3% Fluorescent Brightener 28 [69]. Calcofluor-stained hyphae were observed by fluorescence binocular microscopy, or a Zeiss Axioplan 2 fluorescence microscope configured for brightfield, darkfield, and differential interference contrast (excitation 480 nm, barrier filter 510 nm).

### RNA methods

Total RNA was extracted from *A. thaliana* seedlings using TRIZOL Reagent (Invitrogen) following the instructions of the manufacturer. One µg RNA was reverse-transcribed using the iScript cDNA Synthesis Kit (Biorad). For *H. arabidopsidis* ITS2 RNA quantification within infected tissues, PCR (25 cycles) was performed with 8 ng cDNA as template using the ITS2 forward primer 5′-TGTGGTAGACGAATGGGTGA-3′, and the ITS2 reverse primer 5′-AAGTGCAGCCGAAGCTTTAC-3′. PCR with the primer pairs OXA1-a and OXA1-b (Figure S1) was used as a quantitative control. Aliquots of individual PCR products were resolved by agarose gel electrophoresis, visualized with ethidium bromide and quantified using a Fujifilm FLA-3000 Phospho/Fluoroimager. The expression of *PR-1a* and *PDF1.2b* was analyzed by real-time quantitative PCR using the forward primer 5′-GGAGCTACGCAGAACAACTAAGA-3′ and reverse primer 5′-CCCACGAGGATCATAGTTGCAACTGA-3′ for *PR-1a*, and the forward primer 5′-TCATGGCTAAGTTTGCTTCC-3′, and reverse primer 5′-AATACACACCACGATTTAGCACC-3′ for *PDF1.2b*. Amplification and detection were performed in the Chromo4 detection system (Biorad). Reactions were done in a final volume of 15 µl containing 10 µl qPCR MasterMix Plus For SYBRGreen I No Rox (Eurogentec), 0.5 mM of each primer, and 8 ng of cDNA template. PCR conditions were as follows: 95°C for 15 min, followed by 40 cycles of 95°C for 15 s, 56°C for 30 s and 72°C for 30 s. At the end of the program a melting curve (from 60°C to 95°C, read every 0.5°C) was determined to ensure that only single products were formed. *Ubiquitin-specific protease 22* (*UBP22*) expression was used to normalize the transcript level in each sample with the primer pairs 5′-GCCAAAGCTGTGGAGAAAAG-3′ and 5′-TGTTTAGGCGGAACGGATAC-3′. Data analysis was performed using the MJ OpticonMonitor Analysis software (version 3.1; Biorad).

### Extraction and analysis of hydroxycinnamoyl malates

Extraction of soluble phenolics from fresh plants was carried out in 100% methanol (0.4 ml/100 mg of fresh weight). After centrifugation, the cleared supernatant was adjusted to 80% aequeous methanol, and passed through a 200 mg C_18_ (Nucleodur 100-30, Macherey-Nagel, Düren, Germany), in order to stop chorophyll and lipidic compounds. The unretained fraction was directly analyzed by HPLC. The liquid chromatograph (System Controller 680 with two 510 pumps; Waters Millipore, Milford, MA) was equipped with an Inertsil 5ODS3 C_18_ column (5 µm, 250×4,6 mm i.d.; Interchim, Montluçon, France). Samples were chromatographied with the following gradient (flow rate l ml/min): 1 min isocratic 15% solvent A (methanol) and 85% solvent B (water, 0.5% H_3_PO_4_), then within 29 min to 75% solvent A, then within 3 additional min to 100% solvent A, followed by a 3 min isocratic step in 100% solvent A. Compounds were detected by a Waters TM 996 Photodiode Array Detector (200 to 500 nm). Peaks were identified and quantified using the Empower software (Waters), after external standardization with synthetic compounds.

### Analysis of oomycete reproduction *in vitro*

The *P. cactorum* isolate 723 was from the Sophia Antipolis *Phytophthora* collection, and was sampled in 2005 from *Fragaria* in France. Propagation and zoospore production were performed as described [70], and 5,000 zoospores were pipetted into 0.5 ml of V8 medium [70] in 24-well titer plates. After 24 h at 24°C, another 0.5 ml of V8 medium containing or not the hydroxycinnamoyl malates were added. After an incubation for further 48 h at 24°C, mycelium was picked out of the wells, rinsed, dried on filter paper, weighed, placed into 1 ml of water, and macerated in a potter. Liberated oospores were enumerated with a hemocytometer and the number of oospores was expressed per g mycelium fresh weight.

### Gene accession numbers

Sequences used in this article were derived from gene IDs 835504 (*COMT1*), 169452 (*PtOMT1*), 1815949 (*PR1-a*), 817143 (*PDF1.2b*), 836325 (*OXA1*), 830946 (*UBP22*).

## Supporting Information

Figure S1 Molecular analyses of the mutants and transgenic lines used in this study.
(0.57 MB PDF)

Figure S2 Susceptibility of COMT1 knock-outs to fungal pathogens and a nematode.
(0.82 MB PDF)

Figure S3 Susceptibility of COMT1 knock-outs to bacterial pathogens.
(0.11 MB PDF)

Figure S4 Comt1a mutants are not altered in SA- and JA-dependent defense responses.
(0.10 MB PDF)

Figure S5 Reversed-phase HPLC of synthetic hydroxycinnamoyl malate esters and of methanolic plant extracts.
(0.30 MB PDF)

Protocol S1 Characteristics of fungal and bacterial pathogens, and inoculation procedures.
(0.03 MB DOC)

Protocol S2 Synthesis and spectral analyses of hydroxycinnamoyl malates.
(0.03 MB DOC)
