# Supplementary material for: Imbalanced Lignin Biosynthesis Promotes the Sexual Reproduction of Homothallic Oomycete Pathogens
Source: PLoS Pathog. 2009 Jan 16;5(1):e1000264. doi: 10.1371/journal.ppat.1000264 (PMC2613516; doi:10.1371/journal.ppat.1000264)
Supplement: Figure S1 — Molecular analyses of the mutants and transgenic lines used in this study. (0.57 MB PDF) [file ppat.1000264.s001.pdf]

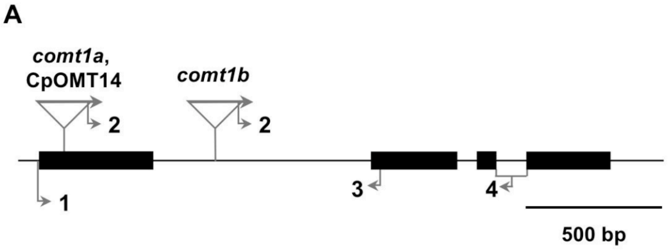

B

Primer	Target	Sequence (5' to 3')
1	*COMT1* (*Arabidopsis* and Poplar; Genomic Sequence and cDNA)	YAARATGGGTTCAACRGSWG
2	T-DNA left Border	CTACAAATTGCCTTTTCTTATCGAC
3	*COMT1* (*Arabidopsis* and Poplar; Genomic Sequence and cDNA)	CCWCCATCAAGAATTGCATC
4	*COMT1* (*Arabidopsis* and Poplar; cDNA)	CATGRCATATCCACTTCATG
OXA1-a	*OXA1* cDNA	TACCTGATCTGCCTCCACCT
OXA1-b	*OXA1* cDNA	AACAGGACTCAGCGATGTTG

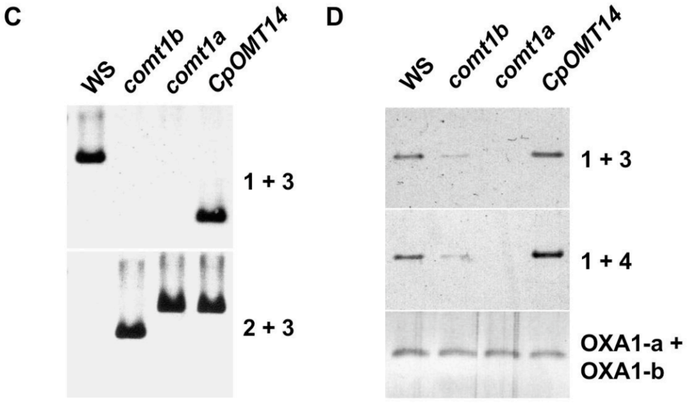

Figure S1. Molecular analyses of the mutants and transgenic lines used in this study. **A.** Schematic illustration of the genomic organization of *COMT1* (locus At5g54160), primer attachment sites, and T-DNA insertion sites and orientations in genomic DNA from lines *comt1a*, *comt1b,* and CpOMT14. Bars represent exons, lines correspond to introns and untranslated sequences. **B.** Names, targets, and sequences of primers used for the analyses. **C.** Primerpair 1 + 3 revealed the presence of an amplicon spanning the first exon and the first intron in wild-type (WS) genomic DNA. The absence of this amplicon in DNA from lines *comt1b*, *comt1a*, and CpOMT14 indicated T-DNA insertions. In line CpOMT14, PCR with primerpair 1 + 3 revealed the integrated *COMT1* cDNA sequence from poplar. Primerpair 2 + 3 detected the integrated T-DNA in lines *comt1a*, *comt1b*, and CpOMT14. Sequencing of the amplicon from line *comt1b* confirmed the insertion site. Genomic DNA was extracted with the Extract-N-Amp Plant PCR kit (Sigma). **D.** RT-PCR revealed *COMT1* transcripts in wild-type *Arabidopsis* and in the CpOMT14 line. The amplicon was absent from the *comt1a* line, but was detectable in extracts from the *comt1b* line. The sequences of the wild-type and *comt1b* amplicons were identical, indicating that *comt1b,* unlike *comt1a,* is not a full knockout mutant line. The T-DNA insertion in intron 1 resulted, however, in reduced levels of *COMT1* mRNA accumulation. Amplification of the constitutively expressed *OXA1* gene (At5g62050) transcript showed that similar amounts of intact cDNAs were used for RT-PCR experiments.
