# Supplementary material for: Imbalanced Lignin Biosynthesis Promotes the Sexual Reproduction of Homothallic Oomycete Pathogens
Source: PLoS Pathog. 2009 Jan 16;5(1):e1000264. doi: 10.1371/journal.ppat.1000264 (PMC2613516; doi:10.1371/journal.ppat.1000264)
Supplement: Figure S2 — Susceptibility of COMT1 knock-outs to fungal pathogens and a nematode. (0.82 MB PDF) [file ppat.1000264.s002.pdf]

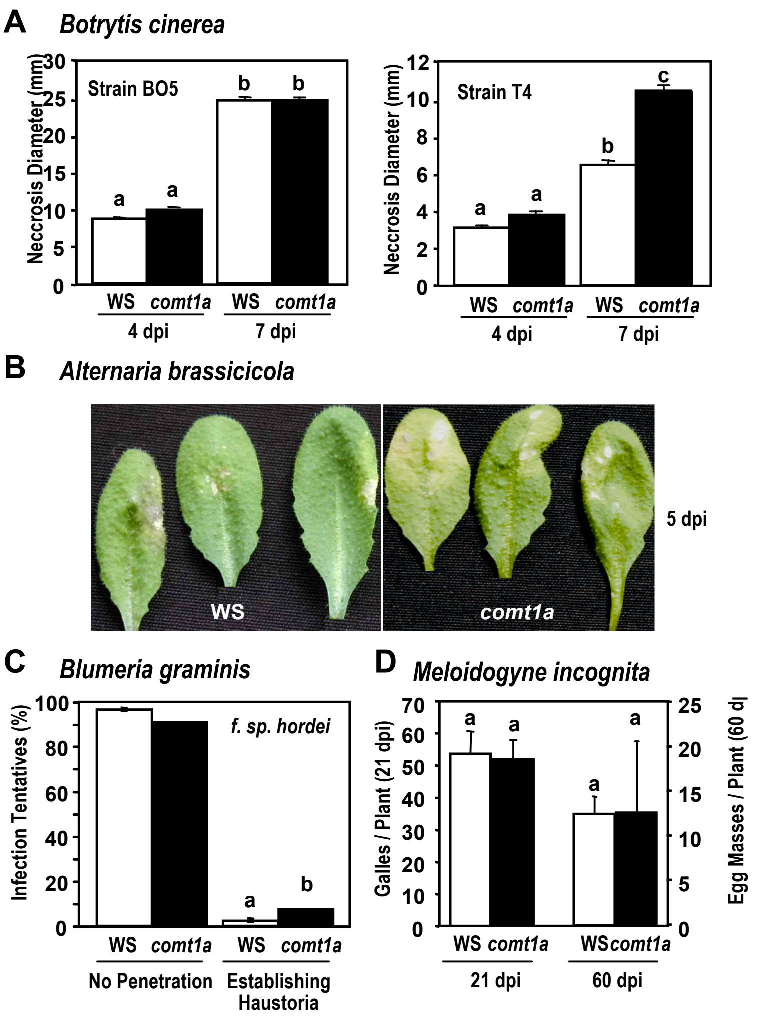

Figure S2. Susceptibility of *COMT1* knock-outs to fungal pathogens and a nematode. **A.** Plants from wild-type Arabidopsis and from the mutant line *comt1a* were similarly susceptible to the aggressive *Botrytis cinerea* strain B05.10 (left). In contrast, when inoculated with the less aggressive strain T4, invasion was increased on the *comt1a* mutant, when compared to the wild-type plants (right). **B.** Leaf inoculations of wild-type *A. thaliana* with *Alternaria brassicicola* resulted in the appearance of necrotic spots, which remained localized to the infection sites and did not affect leaf viability (left). Plants from the *comt1a* background were still resistant to the pathogen in the absence of COMT but the fungus was not restricted to the necrotic inoculation area, and developed outside by generating chlorotic yellow halos (right). **C.** On wild-type Arabidopsis, the majority of *Blumeria graminis f. sp hordei* germlings failed to penetrate the epidermal cell wall and papillae, and successful infection was rather the exception. On the *comt1a* mutant line, the proportion of successful infection was increased. **D.** The knock-out of *COMT1* did not influence the average number of galls per plant that were established by *Meloidogyne incognita* infection. The successful completion of the nematode infection cycle was characterized by the production of eggs. No significant differences in the average number of egg masses per plant were observed, when comparing infected wild-type and mutant plants. Bars represent mean values ± SD from at least 3 experiments. Statistically significant differences for values compared among each other were determined by Student's t-test (identical lettering, not significantly different with P > 0,01; different lettering, P < 0.001; double lettering P < 0,01).
