# Supplementary material for: Imbalanced Lignin Biosynthesis Promotes the Sexual Reproduction of Homothallic Oomycete Pathogens
Source: PLoS Pathog. 2009 Jan 16;5(1):e1000264. doi: 10.1371/journal.ppat.1000264 (PMC2613516; doi:10.1371/journal.ppat.1000264)
Supplement: Figure S3 — Susceptibility of COMT1 knock-outs to bacterial pathogens. (0.11 MB PDF) [file ppat.1000264.s003.pdf]

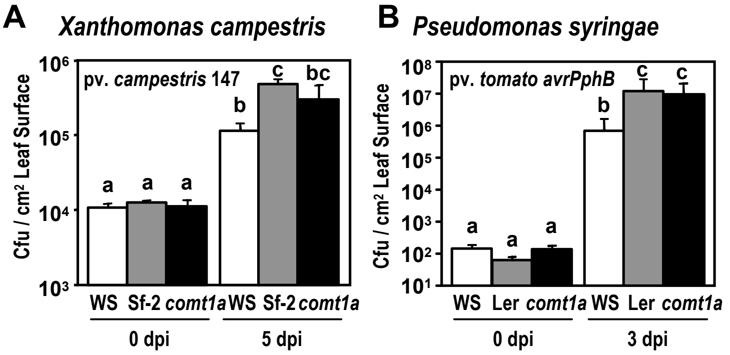

Figure S3. Susceptibility of *COMT1* knock-outs to bacterial pathogens.
A. The *comt1a* mutant revealed to be significantly more susceptible to *Xanthomonas campestris* pv. *campestris* than the WS wild-type background, but was not as susceptible as the Sf-2 ecotype. **B.** *Pseudomonas syringae* pv. *tomato* strain DC3000 carrying the *P. syringae* pv. *phaseolicola* avirulence gene *avrPphB* is recognized by RPS5 present in the WS background, but lacking in Ler [45]. Inoculations with *Pst avrPphB* revealed that the mutation of *COMT1* renders WS as susceptible as Ler. Bars represent mean values ± SD from at least 3 experiments. Statistically significant differences for values compared among each other were determined by Student's t-test (identical lettering, not significantly different with P > 0,01; different lettering, P < 0.001; double lettering P < 0,01).
