# Supplementary material for: Imbalanced Lignin Biosynthesis Promotes the Sexual Reproduction of Homothallic Oomycete Pathogens
Source: PLoS Pathog. 2009 Jan 16;5(1):e1000264. doi: 10.1371/journal.ppat.1000264 (PMC2613516; doi:10.1371/journal.ppat.1000264)
Supplement: Figure S4 — Comt1a mutants are not altered in SA- and JA-dependent defense responses. (0.10 MB PDF) [file ppat.1000264.s004.pdf]

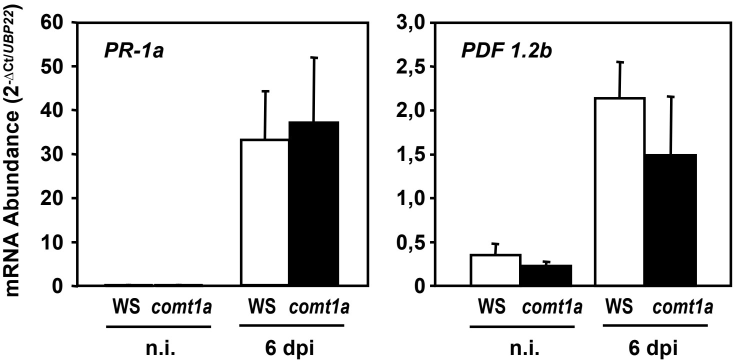

Figure S4. *Comt1a* mutants are not altered in SA- and JA-dependent defense responses. Quantitative RT-PCR experiments were performed with gene-specific primers for *PR-1a* (locus At2g14610) and *PDF1.2b* (At2g26020), on cDNA populations corresponding to transcripts accumulating in non-inoculated plants (n.i.), and 6 days post-inoculation (dpi) with *H. arabidopsidis* isolate Emwa1. Ct values were normalized for input cDNA with data obtained for the constitutively expressed *UBP22* gene (At5g10790). The bars indicate mean values ± SD from 2 independent experiments.
