# Supplementary material for: Imbalanced Lignin Biosynthesis Promotes the Sexual Reproduction of Homothallic Oomycete Pathogens
Source: PLoS Pathog. 2009 Jan 16;5(1):e1000264. doi: 10.1371/journal.ppat.1000264 (PMC2613516; doi:10.1371/journal.ppat.1000264)
Supplement: Figure S5 — Reversed-phase HPLC of synthetic hydroxycinnamoyl malate esters and of methanolic plant extracts. (0.30 MB PDF) [file ppat.1000264.s005.pdf]

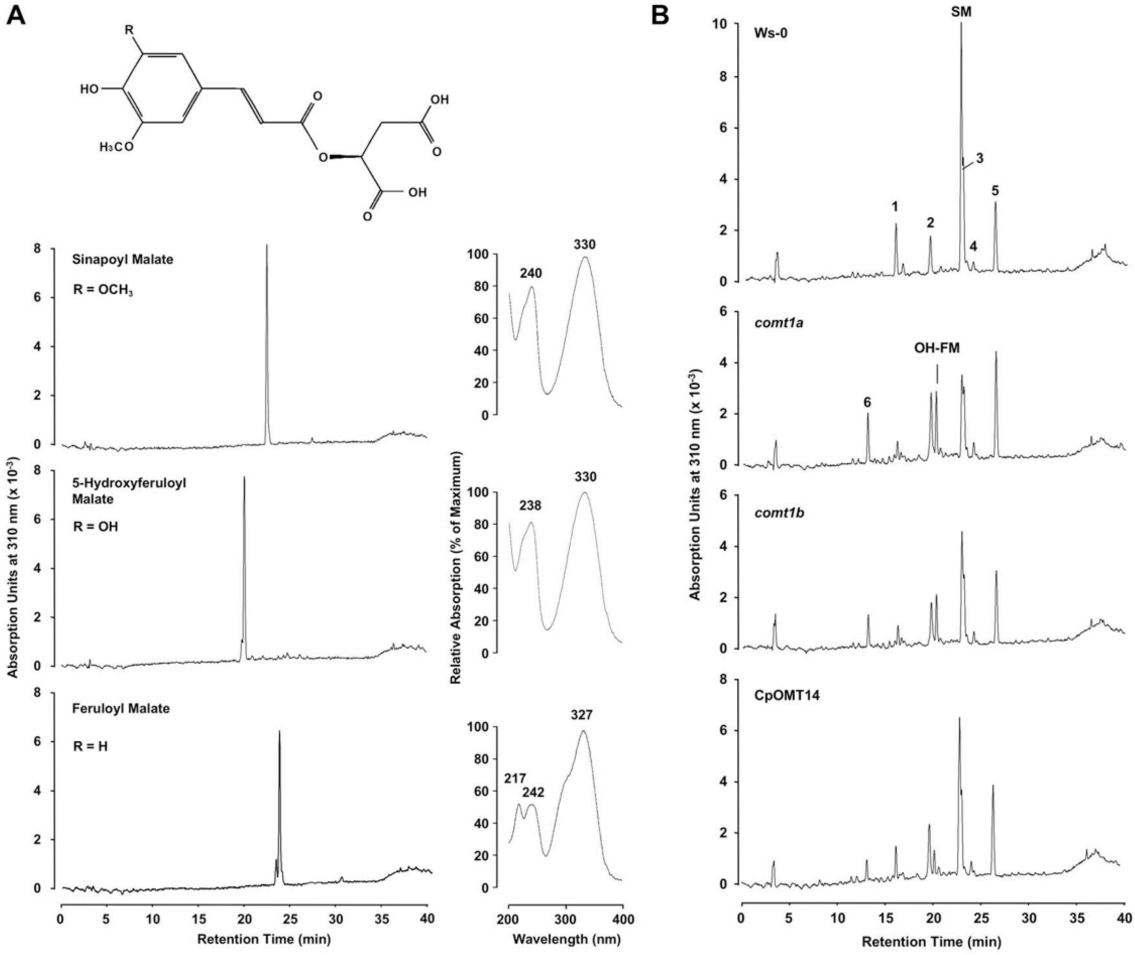

Figure S5. Reversed-phase HPLC of synthetic hydroxycinnamoyl malate esters and of methanolic plant extracts. **A.** Chemical structures, elution profiles and UV absorption spectra for sinapoyl malate (SM), 5-hydroxyferuloyl malate (OH-FM), and feruloyl malate (FM). The chromatograms represent 15 pmol, 30 pmol, and 32 pmol of the esters, respectively. **B.** Elution profiles of compounds extractable with aqueous methanol (80 %) from 4-week-old plantlets of wild-type *A. thaliana* (WS), the two allelic *comt1* mutant lines (*comt1a* and *comt1b*), and the complemented mutant line (CpOMT14). SM and OH-FM were identified and quantified by co-chromatography and spectral analysis with synthetic standards used for reference. Spectral absorption data suggested that the compounds marked 1 and 6 were additional esters of sinapic acid and 5-hydroxyferulic acid, respectively. The compounds marked 2 to 5 represent flavonoids or flavonoid glycosides. Compounds 1 to 6 were not characterized further.
