# Supplementary material for: Imbalanced Lignin Biosynthesis Promotes the Sexual Reproduction of Homothallic Oomycete Pathogens
Source: PLoS Pathog. 2009 Jan 16;5(1):e1000264. doi: 10.1371/journal.ppat.1000264 (PMC2613516; doi:10.1371/journal.ppat.1000264)
Supplement: Protocol S1 — Characteristics of fungal and bacterial pathogens, and inoculation procedures. (0.03 MB DOC) [file ppat.1000264.s006.doc]

**Protocol S1 Supporting Materials and Methods**

**Characteristics of fungal and bacterial pathogens, and inoculation procedures.**

***Botrytis cinerea***

*Botrytis cinerea* is a necrotrophic fungus able to infect a wide range of plants by killing host tissues. Following the inoculation of leaves on *A. thaliana* plants with the highly aggressive strain B05.10 [24], the necrotic lesion phenotype extended to almost the entire leaf, regardless of the genotype of the plant (Supplementary Figure S2A, left). By contrast, after inoculation with the less aggressive *B. cinerea* strain T4 [24], invasion levels were 60 % higher on the *comt1* mutant than on the wild-type plants (Supplementary Figure S2A, right). Pathogenicity assays were performed as previously described [71], except that the final concentration was 1 x 10^6^ conidia/ml. Excised *Arabidopsis* leaves were inoculated with 10 µl droplets of conidial suspensions. Disease development was determined 4 and 7 dpi, by measuring radial spread from the point of inoculation to the margin of the lesion. Pathogenicity assays were repeated nine times, and each repetition comprised ten independent measurements of lesion size per strain.

***Alternaria brassicicola***

All natural accessions of *A. thaliana* are resistant to the necrotrophic fungal pathogen, *Alternaria brassicicola*. In mutants with impaired resistance signaling pathways [25], disease caused by *Alternaria brassicicola* appears on the leaves as necrotic lesions surrounded by chlorotic yellow halos. In our experiments, leaf inoculations of wild-type *A. thaliana* with the fungus resulted in the appearance of necrotic spots, which remained restricted to the infection sites and did not affect leaf viability (Supplementary Figure S2B, left). Mutant *comt1a* plants were resistant to the pathogen, but the fungus was not restricted to the necrotic inoculation area, leading to the generation of characteristic chlorotic yellow halos (Supplementary Figure S2B, right). *Alternaria brassicicola* was propagated and spores were harvested as previously described [72,73]. Five-week-old wild-type and mutant plants were inoculated with 5 µl droplets of a 1 x 10^6^ spores/ml suspension. Plants were incubated in a growth chamber at high humidity. The lid was removed 3 dpi, and disease symptoms were analyzed 5 dpi.

***Blumeria graminis* *f. sp* *hordei (Bgh)***

The inoculation of wild-type *A. thaliana* with the biotrophic fungal pathogen *Bgh* had no visible effect. Most germlings failed to penetrate the epidermal cell wall and papillae, and successful infection was rare (Supplementary Figure S2C). A similar pattern was observed with the *comt1a* mutant line, except that the proportion of infections that were successful was at least twice as high (Supplementary Figure S2C). *Bgh* was cultured on the susceptible barley cultivar Ingrid Mlo as previously described [74-76]. Four-week-old wild-type and mutant plants were placed in a 2 m high settling tower and inoculated by dusting the conidia from infected barley leafs at the top of the tower [26]. After 1 h, the plants were returned to the growth chamber. Three dpi, leaves were harvested and stained with trypan blue, as previously described [76], to visualize conidia and *Bgh* growth. For every genotype, three leaves with 100 interaction sites were counted and the data were analyzed with SigmaStat.

***Xanthomonas campestris* pathovar *campestris (Xcc)*** **and** ***Pseudomonas syringae* pv. *tomato (Pst)***

*Xcc* causes black rot in crucifers, including *A. thaliana*. This bacterium is a xylem-colonizing systemic pathogen that generally invades plant leaves through hydathodes and multiplies in vascular tissues [77]. *Arabidopsis* has a generally high level of resistance to *Xcc* strain 147, which varies with ecotype. Upon infiltration into leaves, bacteria multiply more effectively in the Sf-2 background than in the WS background. These two ecotypes are thus considered to be susceptible and resistant, respectively [27]. With this inoculation procedure, the *comt1a* mutant was found to be significantly more susceptible than wild-type WS plants, but was not as susceptible as the Sf-2 ecotype (Supplementary Figure S2E). The *comt1* mutation thus weakened resistance to *Xcc* 147. Similar results were obtained when leaves were inoculated with the bacterial speck pathogen, *Pst* strain DC3000 carrying *avrPphB* (Supplementary Figure S2F). The *Xcc*147 strain was cultured at 28°C on Kado medium [78] supplemented with rifampicin (50 µg/ml) and spectinomycin (50 µg/ml). The *Pst* *avrPphB* strain was cultured at 28 °C on King B medium supplemented with 50 µg/ml rifampicin and 20 µg/ml kanamycin. For all experiments with *Xcc* and *Pst*, mutant and wild-type seeds were sterilized and sown as previously described [79]. Seedlings were transferred to Jiffy pots and grown in a growth chamber under a nine-hour photoperiod, at 21 °C and 40 to 70 % humidity. Four- or five-week-old plants were used for bacterial inoculation. They were kept at high humidity for the 12 hours preceding the experiments and were then grown under a nine-hour photoperiod, 21 °C and 90 % humidity. Leaves were infiltrated with bacterial suspensions of 1 x 10^5^ cfu/ml and 1 x 10^8^ cfu/ml for *Pst* and *Xcc*, respectively. Bacterial growth *in planta* was assessed as previously described [80].

**Supplemental References**

71. Viaud M, Brunet-Simon A, Brygoo Y, Pradier JM, Levis C (2003) Cyclophilin A and calcineurin functions investigated by gene inactivation, cyclosporin A inhibition and cDNA arrays approaches in the phytopathogenic fungus *Botrytis cinerea*. Mol Microbiol 50:1451-1465.

72. Penninckx IA, Eggermont K, Terras FR, Thomma BP, De Samblanx GW, *et al.* (1996) Pathogen-induced systemic activation of a plant defensin gene in *Arabidopsis* follows a salicylic acid-independent pathway. Plant Cell 8:2309-2323.

73. Thomma BP, Nelissen I, Eggermont K, Broekaert WF (1999) Deficiency in phytoalexin production causes enhanced susceptibility of *Arabidopsis thaliana* to the fungus *Alternaria brassicicola*. Plant J 19:163-171.

74. Jarosch B, Kogel KH, Schaffrath U (1999) The ambivalence of the barley *Mlo* locus: mutations conferring resistance against powdery mildew (*Blumeria graminis* f. sp. *hordei*) enhance susceptibility to the rice blast fungus *Magnaporthe grisea*. Mol Plant Microbe Interact 12:508-514.

75. Jarosch B, Collins NC, Zellerhoff N, Schaffrath U (2005) RAR1, ROR1, and the actin cytoskeleton contribute to basal resistance to *Magnaporthe grisea* in barley. Mol Plant Microbe Interact 18:397-404.

76. Jansen M, Jarosch B, Schaffrath U (2007) The barley mutant *emr1* exhibits restored resistance against *Magnaporthe oryzae* in the hypersusceptible *mlo*-genetic background. Planta 225:1381-1391.

77. Alvarez AM (2000) in *Mechanisms of Resistance to Plant Disease*, eds Slusarenko AJ, Fraser RSS, Van Loon LC (Kluwer, Dordrecht), pp 21-52.

78. Kado CI, Heskett MG (1970) Selective media for isolation of *Agrobacterium*, *Corynebacterium*, *Erwinia*, *Pseudomonas*, and *Xanthomonas*. Phytopathol 60:969-976.

79. Balagué C, Lin B, Alcon C, Flottes G, Malmström S, *et al.* (2003) HLM1, an essential component of signaling for the Hypersensitive Response, encodes a member of the CNGC ion channel family. Plant Cell 15:365-379.

80. Lorrain S, Lin B, Auriac MC, Kroj T, Saindrenan P, *et al.* (2004) VASCULAR ASSOCIATED DEATH 1, a novel GRAM domain-containing protein, is a regulator of cell death and defense responses in vascular tissues. Plant Cell 16:2217-2232.
