# Supplementary material for: Imbalanced Lignin Biosynthesis Promotes the Sexual Reproduction of Homothallic Oomycete Pathogens
Source: PLoS Pathog. 2009 Jan 16;5(1):e1000264. doi: 10.1371/journal.ppat.1000264 (PMC2613516; doi:10.1371/journal.ppat.1000264)
Supplement: Protocol S2 — Synthesis and spectral analyses of hydroxycinnamoyl malates. (0.03 MB DOC) [file ppat.1000264.s007.doc]

**Protocol S2 Supporting Materials and Methods**

**Synthesis of hydroxycinnamoyl malates.**

All moisture and O_2_-sensitive reactions were carried out in flame-dried glassware under N_2_. Evaporations were conducted under reduced pressure at temperatures below 45 °C unless otherwise noted. Column chromatography (CC) was carried out under positive N_2_ pressure with 40-63 µm silica gel (Merck) and the indicated solvents. Chemicals were bought from Sigma-Aldrich and Acros Organics and used as received.

Synthesis of the di-*t*-butylmalate ester: To a suspension of malic acid (3.6 g, 29.0 mmol, 1 equiv.) in CH_2_Cl_2_ (300 ml) at room temperature (rt) under argon, *N*,*N’*-diisopropyl-*O*-*t*-butylisourea (38.8 g, 193.7 mmol, 6.7 equiv.) was added, and the solution was stirred for 2 days at rt. The crude mixture was concentrated in *vacuo*, cyclohexane (400 ml) was added, and the resulting mixture was filtered over Celite. The filtrate was concentrated in *vacuo* and the crude oil was purified by flash chromatography (Cyclohexane:AcOEt = 5:5) to yield di-*t*-butylmalate ester as a yellow oil (4.49 g, 63% yield). The Steglich esterification of acetylated ferulic acid and di-t-Butyl-malate ester provided the protected malates. To a solution of 4-acetoxy-ferulic acid (5 g, 21.16 mmol, 1 equiv.), di-*t*-butylmalate ester (5.2 g, 21.16 mmol, 1 equiv.) in CH_2_Cl_2_ (106 ml) at rt, diisopropylcarbodiimide (DIC; 3.33 ml, 2.67 g, 21.16 mmol, 1 equiv.), and 4-(dimethylamino)pyridine (DMAP; 2.58 g, 10.58 mmol, 0.5 equiv.) were added. The mixture was stirred at rt for 1 day. The crude mixture was poured into hexane and the resulting mixture was filtered over Celite. The filtrate was concentrated in *vacuo* and the crude solid was purified by flash chromatography (Cyclohexane:AcOEt = 7:3) to yield the protected feruloyl malate as a white solid (8.2 g, 84% yield). Protected feruloyl malate ester (410 mg, 0.88 mmol, 1 equiv.) was then dissolved in CH_2_Cl_2_ (4 mL) at rt, and 99% TFA (1.3 mL, 2 g, 17.7 mmol, 20 equiv.) was added dropwise. The solution was stirred overnight and concentrated in *vacuo* to afford the free diacid as a crude oil which was used in the next step without further purification (287.1 mg, 93% yield). To a mixture of the free diacid (309 mg, 0,88 mmol, 1 equiv.) in acetone (30 ml), 3N HCl_aq_ (9 ml) was added. The resulting solution was refluxed for 3 hours, cooled to rt and diluted with ethyl acetate (200 ml). The solution was then washed with brine, dried over MgSO_4_, and concentrated in *vacuo* to afford feruloyl malate as a crude oil which was recrystallized in water to give a white solid (266.5 mg, 98% yield). Sinapoyl malate and 5-hydroxyferuloyl malate were obtained by the same procedure, using acetylated sinapic acid and acetylated 5-hydroxyferulic acid in the Steglich esterification.

**Spectral analyses of hydroxycinnamoyl malates.** ^1^H Spectra of samples in the indicated solvent were recorded at 300 MHz on a MercuryPlus 300 Varian instrument (^1^H NMR: CDCl_3_ residual signal at 7.26 ppm; CD_3_OD residual signal at 3.31 ppm; (CD_3_)_2_CO residual signal at 2.05 ppm). ^13^C NMR Spectra of samples in the indicated solvent were recorded at 75 MHz on a Varian instrument (^13^C NMR: CDCl_3_ residual signal at 77.26 ppm; CD_3_OD residual signal at 49.00 ppm; (CD_3_)_2_CO residual signal at 29.84 ppm). ^13^C multiplicities were determined by DEPT_135_ experiments. Infrared analyses were performed on a Nicolet *Avatar 320 FT-IR*. Optical rotations were measured on a *Bellingham+ ADP410* from Stanley. Electron impact (EI), and low- and high-resolution (HR) MS analyses were obtained from the mass spectrometry service of the ICSN-CNRS – Gif-sur-Yvette, France. Elemental analyzes were obtained from the ICSN-CNRS – Gif-sur-Yvette, France.

2-*O*-feruloyl-L-malate: [α]_D_^26^ + 10.0 (c 0.002, CH_3_OH). IR (neat): 3314, 2974, 1708, 1046 cm^-1^. ^1^H NMR (300 MHz, CD_3_OD): d = 7.63 (d, *J* = 15.9 Hz, 1H, H-7’), 7.20 (s, 1H, H-2’), 7.05 (d, *J* = 8.1 Hz, 2H, H-6’), 6.79 (d, *J* = 8.1 Hz, 1H, H-5’), 6.38 (d, *J* = 15.9 Hz, 1H, H-8’), 5.49 (dd, *J* = 3.6 and 8.1 Hz, 1H, H-2), 3.86 (s, 3H, *CH_3_*O-3’), 3.01 (dd, *J* = 3.6 and 15.9 Hz, 1H, H-3), 2.89 (dd, *J* = 8.1 and 15.9 Hz, 1H, H-3). ^13^C NMR (75 MHz, CD_3_OD): d = 172.9 (s, C1), 172.5 (s, C4), 168.0 (s, C9), 150.7 (s, C4’), 149.3 (s, C3’), 147.8 (d, C7’), 127.5 (s, C1’), 124.3 (d, C6’), 116.4 (d, C5’), 114.5 (d, C8’), 111.7 (d, C2’), 69.9 (d, C2), 56.4 (q, *CH_3_*O-3’), 37.0 (t, C3). Sinapoyl-L-malate: [α]_D_^26^ + 40.0 (c 0.002, CH_3_OH). IR (neat): 3340, 2975, 1718, 1044, 879 cm^-1^. ^1^H NMR (300 MHz, CD_3_OD): d = 7.60 (d, *J* = 15.9 Hz, 1H, H-7’), 6.86 (s, 2H, H-2’, H-6’), 6.40 (d, *J* = 15.9 Hz, 1H, H-8’), 5.49 (dd, *J* = 3.6 and 8.1 Hz, 1H, H-2), 3.83 (s, 6H, *CH_3_*O-3’, *CH_3_*O-5’), 3.01 (dd, *J* = 3.6 and 15.9 Hz, 1H, H-3), 2.89 (dd, *J* = 8.1 and 15.9 Hz, 1H, H-3). ^13^C NMR (75 MHz, CD_3_OD): d = 172.9 (s, C1), 172.5 (s, C4), 168.0 (s, C9), 149.3 (s, C3’, C5’), 148.0 (d, C7’), 139.6 (s, C4’), 126.4 (s, C1’), 115.0 (d, C8’), 107.0 (d, C2’, C6’), 69.9 (d, C2), 56.8 (s, *CH_3_*O-3’, *CH_3_*O-5’), 37.0 (t, C3). 5-Hydroxyferuloyl-L-malate: [α]_D_^26^ + 55.0 (c 0.002, CH_3_OH). IR (neat): 2975, 1726, 1264, 1171, 1095 cm^-1^. ^1^H NMR (300 MHz, (CD_3_)_2_CO): d = 9.69 (bs, 2H, CO_2_*H* x 2), 7.65 (d, *J* = 15.9 Hz, 1H, Ar-*CH*=CH-CO_2_R), 7.35 (s, 1H, H-x’), 7.12 (s, 1H, H-x’), 6.61 (d, *J* = 15.9 Hz, 1H, Ar-CH=*CH*-CO_2_R), 5.43 (dd, *J* = 3.6 and 8.1 Hz, 1H, H-2), 3.86 (s, 3H, *CH_3_*O-3’), 3.02 (dd, *J* = 3.6 and 16.8 Hz, 1H, H-3), 2.92 (dd, *J* = 8.1 and 16.8 Hz, 1H, H-3), 2.23 (s, 6H, *CH_3_*CO_2_-4’, *CH_3_*CO_2_-5’).^13^C NMR (75 MHz, CDCl_3_): d = 171.0 (s, *C*O_2_H), 170.6 (s, *C*O_2_H), 168.7 (s, CH*_3_C*O_2_-4’), 167.9 (s, CH_3_*C*O_2_-5’), 166.1 (s, *C*-9), 153.8 (s, *C*-3’), 145.2 (s, *C*-5’), 144.9 (d, *C*-7’), 134.8 (s, *C*-1’), 133.4 (s, *C*-4’), 119.1 (d, *C*-8’), 116.6 (d, *C*-6’), 110.0 (d, *C*-2’), 69.4 (d, CO_2_*CH*CO_2_H), 56.8 (q, *CH_3_*O-3’), 36.4 (t, *CH_2_*CO_2_H), 20.4 (q, *CH_3_*CO_2_-4’), 20.1 (q, *CH_3_*CO_2_-5’).
